# Increased Sensitivity to Chemotherapy Induced by CpG-ODN Treatment Is Mediated by microRNA Modulation

**DOI:** 10.1371/journal.pone.0058849

**Published:** 2013-03-06

**Authors:** Loris De Cecco, Martina Berardi, Michele Sommariva, Alessandra Cataldo, Silvana Canevari, Delia Mezzanzanica, Marilena V. Iorio, Elda Tagliabue, Andrea Balsari

**Affiliations:** 1 Functional Genomics Core Facility, Fondazione Istituto Di Ricovero e Cura a Carattere Scientifico Istituto Nazionale dei Tumori, Milan, Italy; 2 Molecular Targeting Unit, Fondazione Istituto Di Ricovero e Cura a Carattere Scientifico Istituto Nazionale dei Tumori, Milan, Italy; 3 Department of Biomedical Sciences for Health, University of Milan, Milan, Italy; 4 Molecular Therapies Unit, Fondazione Istituto Di Ricovero e Cura a Carattere Scientifico Istituto Nazionale dei Tumori, Milan, Italy; 5 Start Up Unit, Fondazione Istituto Di Ricovero e Cura a Carattere Scientifico Istituto Nazionale dei Tumori, Milan, Italy; The Ohio State University, United States of America

## Abstract

We recently reported that peritumoral CpG-ODN treatment, activating TLR-9 expressing cells in tumor microenvironment, induces modulation of genes involved in DNA repair and sensitizes cancer cells to DNA-damaging cisplatin treatment. Here, we investigated whether this treatment induces modulation of miRNAs in tumor cells and their relevance to chemotherapy response. Array analysis identified 20 differentially expressed miRNAs in human IGROV-1 ovarian tumor cells from CpG-ODN-treated mice versus controls (16 down- and 4 up-regulated). Evaluation of the role of the 3 most differentially expressed miRNAs on sensitivity to cisplatin of IGROV-1 cells revealed significantly increased cisplatin cytotoxicity upon ectopic expression of hsa-miR-302b (up-modulated in our array), but no increased effect upon reduced expression of hsa-miR-424 or hsa-miR-340 (down-modulated in our array). Accordingly, hsa-miR-302b expression was significantly associated with time to relapse or overall survival in two data sets of platinum-treated ovarian cancer patients. Use of bio-informatics tools identified 19 mRNAs potentially targeted by hsa-miR-302b, including HDAC4 gene, which has been reported to mediate cisplatin sensitivity in ovarian cancer. Both HDAC4 mRNA and protein levels were significantly reduced in IGROV-1 cells overexpressing hsa-miR-302b. Altogether, these findings indicate that hsa-miR-302b acts as a “chemosensitizer” in human ovarian carcinoma cells and may represent a biomarker able to predict response to cisplatin treatment. Moreover, the identification of miRNAs that improve sensitivity to chemotherapy provides the experimental underpinning for their possible future clinical use.

## Introduction

Oligodeoxynucleotides (ODN) containing dinucleotides with unmethylated CpG motifs (CpG-ODN) are potent activators of both the innate and adaptive immune systems [1]; [2]. Recognition of CpG-ODN is mediated by Toll-like receptor 9 (TLR9), an endosomal member of the TLR family, which is critically important in detecting microbial pathogens. In a xenograft model of human IGROV-1 ovarian cancer, we recently showed that treatment with CpG-ODN induced down-modulation of DNA repair genes in tumor cells and that peritumoral injection of CpG-ODN in the peritoneal cavity was for inducing this down-modulation [3] and for the antitumor activity of CpG-ODN [4]. Considering the CpG-ODN species specificity and to the lack of TLR9 expression on IGROV1 cells, the effect cannot be mediated by a direct interaction between the oligonucleotide and tumor cells, instead it is likely that peritumoral TLR9-expressing cells, such as innate immune cells and/or endothelial cells, fibroblasts and epithelial cells, directly respond to CpG-ODN and down-regulate DNA repair in tumor cells through a direct cell-cell interaction and/or by secreting soluble factors.

MicroRNAs (miRNAs) are short (∼22 nucleotide), non-coding RNAs known to alter gene expression at the post-transcriptional level [5]; [6]. More than 1,200 human miRNAs have been identified and validated to date (www.mirbase.org), and are predicted to regulate about one-third of the human genome, with involvement in development and progression of many diseases [7]–[9]. Presumably, miRNAs evolved to allow organisms and cells to effectively deal with stress [10]. Recent studies demonstrating that a single miRNA can impact hundreds of targets [11] and that multiple miRNAs can affect a single target [12] point to broad implications of miRNAs, able to affect all most important cellular processes. Indeed, several experimental and clinical findings have also implicated miRNAs in the response to chemotherapy [13], demonstrating a role for miRNAs in the modulation of genes involved in DNA repair [14]; [15].

The CpG-ODN-induced down-modulation of DNA repair genes in tumor cells might represent a physiologic phenomenon that occurs locally in the presence of an infectious event. Upon detection of an infectious agent via endosomal TLRs, cells involved in the immune response might induce modulation of DNA repair genes in infected (or transformed) cells to facilitate their death [16]. Identification of miRNAs that are used “physiologically” to modulate DNA repair genes may have therapeutic implications.

In the present study, we analyzed the effect of CpG-ODN on modulation of miRNAs in tumor cells, the integration of miRNA with mRNA expression modulation induced by CpG-ODN, and the relevance of the identified miRNAs for the response to chemotherapy.

## Materials and Methods

### Ethical Commitee

The *in vivo* experiment was approved by the Ethics Committee for Animal Experimentation of the Fondazione IRCCS Istituto Nazionale Tumori of Milan according to institutional guidelines.

### Drugs and Antibodies

Purified phosphorothioated ODN1826 (5′-TCCATGACGTTCCTGACGTT-3′) containing CpG motifs was synthesized by TriLink Biotechnologies (San Diego, CA, USA). Phosphorothioate modification was used to reduce susceptibility of the ODN to DNase digestion, thereby significantly prolonging its *in vivo* half-life. Cisplatin was purchased from Teva Italia (Milan, Italy). Anti-HDAC4 (D15C3), anti-p21 (sc-397) and anti-GAPDH (GAPDH-71.1) antibodies were purchased from Cell Signaling Technology (Danvers, MA, USA), Santa Cruz Biotechnology (Santa Cruz, CA, USA) and Sigma (St. Louis, MO, USA), respectively.

### Cells

Human IGROV-1 ovarian tumor cells (gift from Dr. J. Benard, Institute Gustave Roussy, Villejuif, France) were adapted to growth i.p. and maintained by serial i.p. passage of ascitic cells into healthy mice as described [3], [17]. Every 6 months, cells were authenticated by morphologic inspection and by FACS analysis for the presence of specific markers. For *in vitro* experiments, IGROV-1 cells were maintained in RPMI medium 1640 supplemented with 10% FCS (Sigma) and 2 mM glutamine (Cambrex, East Rutherford, NJ, USA) at 37°C in a 5% CO_2_ air atmosphere.

### miRNA Extraction from Tumor Samples

miRNAs were extracted from the IGROV-1 xenograft tumors used for gene expression analysis or from a replica of the *in vivo* experiment [3]. Briefly, solid i.p. masses were mechanically disrupted and homogenized in the presence of QIAzol Lysis reagent (Qiagen, Valencia, CA, USA) using a Mikrodismembrator (Braun Biotech International, Melsungen, Germany). RNA was extracted using the miRNeasy Mini kit (Qiagen) according to the manufacturer’s instructions. RNA concentrations were measured with the NanoDrop ND-100 Spectrophotometer (NanoDrop Technologies, Wilmington, DE, USA), while RNA quality was assessed with the Agilent 2100 Bioanalyzer (Agilent Technologies, Palo Alto, CA USA) using the RNA 6000 Nano kit (Agilent). Samples included in the present analysis had a RIN (RNA Integrity Number) score >7 and a 28S:18S rRNA ratio ∼2∶1.

### miRNA Expression Profiling

Mature miRNAs were detected with the Illumina Human_v2 MicroRNA expression profiling kit, based on the DASL (cDNA-mediated Annealing, Selection, Extension, and Ligation) assay, according to the manufacturer’s instructions (Illumina Inc., San Diego, CA, USA). Briefly, 600 ng/sample total RNA was converted to cDNA followed by annealing of a miRNA-specific oligonucleotide pool consisting of: i) a universal PCR priming site at the 5′ end; ii) an address sequence complementary to a capture sequence on the BeadArray; and iii) a miRNA-specific sequence at the 3′ end. After PCR amplification and fluorescent labeling, probes were hybridized on Illumina miRNA BeadChips, washed, and fluorescent signals were detected by the Illumina BeadArrayTM Reader. Data were collected using BeadStudio V3.0 software. Raw and normalized data are available on the Gene Expression Omnibus website with accession numbers GSE41783 and GSE23441 for miRNA and gene expression profiling, respectively.

### Real-time Quantitative PCR (RT-qPCR)

RT-qPCR microRNA assays specific for hsa-miR-18a, hsa-miR-18b, hsa-miR-140-5p, hsa-miR-101, hsa-miR-556-3p, hsa-miR-424, hsa-miR-136, hsa-miR-340, hsa-miR-302b were purchased from Exiqon (Vedbaek, Denmark). RT-qPCR was performed using the miRCURY LNA Universal RT microRNA PCR system (Exiqon) according to the manufacturer’s instructions. Total RNA (20 ng) was polyadenylated and reverse-transcribed at 42°C (60 min), followed by heat-inactivation at 85°C (5 min) using a poly-T primer containing a 5′ universal tag. The resulting cDNA was diluted 80-fold and 8 µl used in 20-µl PCR amplification reactions at 95°C for 10 min, 40 cycles of 95°C for 10 sec, and 60°C for 60 sec. Results were normalized with snord48 (Assay ID:203903). P-values were calculated using two-tailed Student’s t-test.

### Bioinformatics Analysis

Analyses were performed using BRB-Array Tools v4.0 stable release developed by Dr. Richard Simon (NCI) and the BRB-Array Tools development team (EMMES Corp.) and the R package (http://www.bioconductor.org/). The same data-processing was used in both miRNA and gene expression profiling to improve data integration. Quantile normalization was used to correct experimental distortions. A detection threshold of p<0.05 was set for each gene and miRNA. Probes detected in less than 50% of the samples were eliminated from the analysis. Genes and miRNAs differentially expressed were identified using a random-variance t-test, which allows computation of a t-test statistic for each detected miRNA and genes between the classes of samples under investigation without assuming that all miRNAs have the same variance [18]. To limit the number of false-positive findings, miRNAs and genes were considered statistically significant at a false-discovery rate (FDR) <0.1. To identify the most likely targets, mRNA and miRNA expression data were integrated using the MAGIA web tool [19]. A parametric linear correlation measure (Pearson’s correlation coefficient, recommended for normally distributed data and a sample size >5) was used to assess the degree of anti-correlation between miRNA and gene expression data.

### *In silico* Bioinformatics Analyses

Two publicly available datasets GSE27290 [20] and GSE25204 [21] reporting miRNA expression and clinical annotated data were downloaded from the Gene Expression Omnibus (GEO) database. The former dataset consists of 62 diagnosed patients with stage III or IV serous ovarian cancer profiled on a pre-commercial version of miRNA chips (GPL7341) designed on miRBase 9.1. Raw array data were processed using GeneSpring software (Agilent) and quantile-normalized. The latter dataset reports profiling of 85 stage III or IV epithelial ovarian cancers, divided into a training set (55 cases) and test set (30 cases), profiled with Illumina human_v2 MicroRNA chips. Raw data were processed and quantile-normalized using BeadStudio V3.0 software. Non-biological experimental variations between training and test sets were adjusted using ComBat [22].

### Statistical Analysis

The clinical impact on overall survival (OS) and time to relapse (TTR) in GSE27290 and GSE25204, respectively, was assessed by the Kaplan-Meier method, and differences between curves were compared using a non-parametric (log-rank) test, with hazard ratios and 95% confidence intervals also computed. GraphPadPrism v5 (GraphPad software, La Jolla, CA, USA) was used for statistical analyses.

### miRNA Transfection and Cell Viability Analysis

IGROV-1 cells seeded in 6-well plates at 2×10^5^ cells/well were transfected with miRCURY LNA inhibitors of hsa-miR-424 or hsa-miR-340 or negative control A (Exiqon; final concentration, 100 nmol/L) using SiPort Neo-FX (Ambion) according to the manufacturer’s instructions, or with hsa-miR-302b precursor or negative control #1 pre-miR (Ambion; final concentration, 50 nmol/l). Transfections were verified by qRT-PCR as described above. Cell viability after cisplatin treatment were assessed by propidium iodide staining and flow cytometry as described [3].

### Cell Growth Assay

IGROV-1 cells were transfected with 50 nmol/l pre-hsa-miR-302b or scrambled oligonucleotide using SiPort Neo-FX transfection reagent according to the manufacturer’s protocol (Ambion) and seeded in a 96-well plate at a density of 10^3^, 1.5×10^3^, and 2×10^3^ cells/well. After 72 h of culture, cells were fixed with 10% trichloroacetic acid for 1 h at 4°C, washed 5 times with distilled and de-ionized water, air-dried, and incubated with 100 µl sulforodamine (SRB) 0.4% (w/v) for 30 min. Cells were then washed 4 times with 1% acetic acid, air-dried, and 10 mM Tris solution (pH 10.5) added to dissolve the bound dye. Cell growth was assessed based on optical density (OD) at 550 nm using an ELISA microplate reader (Bio-Rad Lab, Inc., Hercules, CA, USA).

### Immunoblotting

Transfected cells were lysed in lysis buffer containing 50 mM Tris-HCl (pH 7.5), 150 mM NaCl, 1% Triton X-100 (Sigma), 10% (vol/vol) glycerol, 2 mM Na-orthovanadate, 10 mM leupeptin, 10 mM aprotinin, 1 mM phenylmethylsulfonyl-fluoride, 100 mM Na-fluoride, and 10 mM Na-pyrophosphate for 30 min at 4°C. Insoluble material was removed by 10-min centrifugation at 15,500 × g at 4°C. Protein concentrations were determined using the Coomassie technique. Equal amounts of total lysates (20 µg) were loaded and separated on 10% precast NuPage SDS-Bis-Tris gels (Invitrogen) and transferred to PVDF membranes (Millipore, Billerica, MA, USA). Western blots were performed with the indicated antibodies, and binding was detected with peroxidase-conjugated secondary antibodies and chemiluminescence ECL (GE Healthcare, Little Chalfont, UK) according to the manufacturer’s instructions. Quantitation of p21 protein levels reportedly regulated post-transcriptionally by miRNAs of the hsa-miR-302 family [23] revealed the expected down-regulation of this protein in IGROV-1 cells transfected with hsa-miR-302b precursor (data not shown), suggesting that hsa-miR-302b exerts biological effects in IGROV-1 cells similar to those observed in other cellular models.

### Plasmid Construction

For luciferase reporter experiments, a 1017-bp region of the HDAC4 3′ untranslated region including the binding site for hsa-miR-302b was amplified from IGROV-1 cells. The PCR product was digested with XbaI and cloned into the reporter plasmid pGL3 control (Promega, Madison, WI, USA) downstream of the luciferase gene. Mutations into the hsa-miR-302b binding site of the HDAC4-3′UTR were introduced using Quik-Change II Site-Directed Mutagenesis kit (Agilent Technologies, Santa Clara, CA).

Primers for Plasmid Construction were:

HDAC4-wt-Fw: 5′-AATTTCTAGAGGGGGACTTAATTCTAATCTCATT-3′.

HDAC4-wt-Rw: 5′-AATTTCTAGATTTTGTGTCAGACCATTACGAA-3′.

HDAC4-Mut-Fw: 5′-GCACTGGCTGGGAGTCAGCAAGCGCCGCGGGTATATCCCTTTGACGGAAACCCTG-3′.

HDAC4-Mut-Rw: 5′-CAGGGTTTCCCTCAAAGGGATATACCCGCGGCGCTTGCTGACTCCCAGCCAGTGC-3′.

### Luciferase Assays for Target and Promoter Identification

pGL3 reporter vector (200 ng) containing the hsa-miR-302b binding site, 40 ng of the phRL-SV40 control vector (Promega), and 50 nmol/l miRNA precursors or scrambled sequence miRNA control (Ambion Inc, Austin, TX. USA) were co-transfected into IGROV-1 cells in 48-well plates. Cells were transfected with Lipofectamine 2000 (Invitrogen) according to the manufacturer’s instructions. Firefly luciferase activity was measured with a Dual Luciferase Assay Kit (Promega) 48 h after transfection and normalized with a Renilla luciferase reference plasmid. Reporter assays were carried out in quadruplicate. Data (mean±S.E.M.) were analyzed using unpaired Student’s t-test.

## Results

### Differentially Expressed miRNAs in IGROV-1 Ovarian Tumors from CpG-ODN-Treated Mice

**Figure 1:**
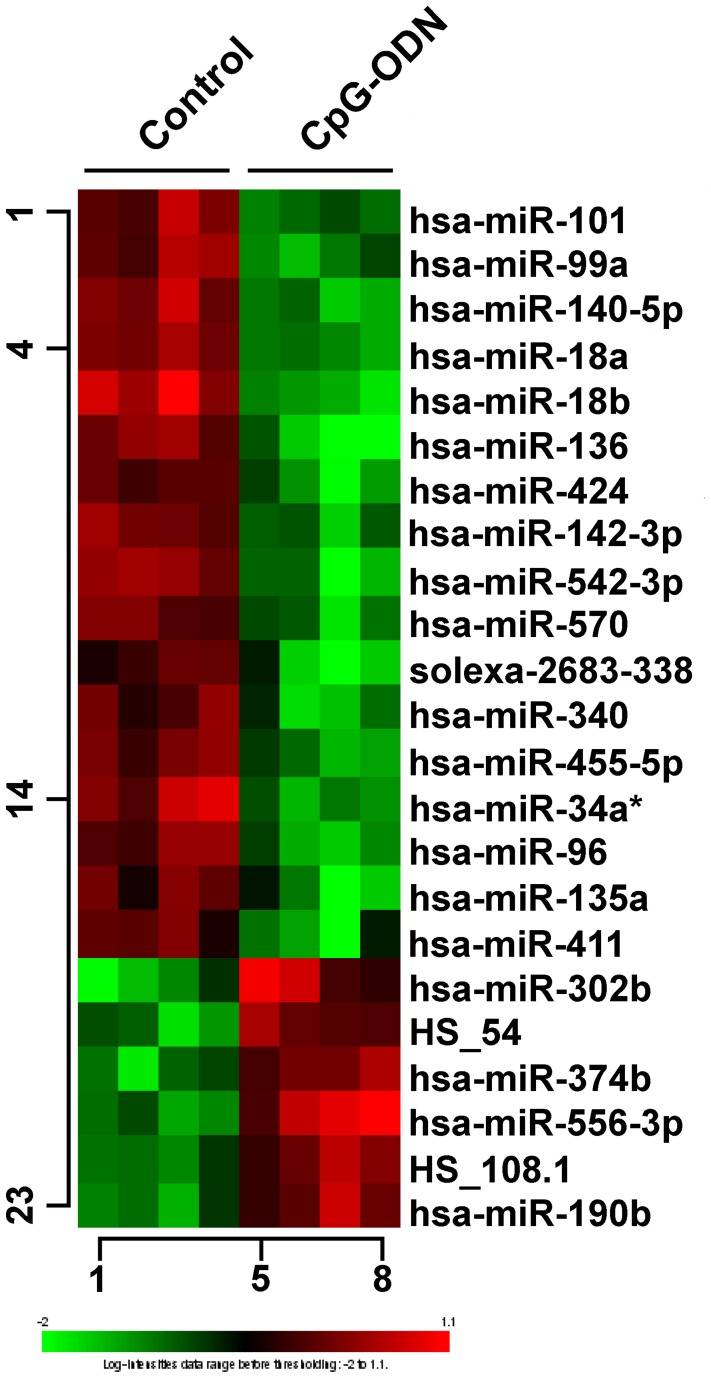
miRNA expression profiling in IGROV-1 ovarian tumors from CpG-ODN-treated athymic mice. Heat-map of 23 modulated miRNAs with FDR <0.1 and fold change >1.8 in CpG-ODN- versus saline-treated mice. Among the 20 miRNAs belonging to miRBase12.0, 16 were down- and 4 up-modulated in CpG-ODN-treated mice (red: up-regulated miRNAs; green: down-modulated miRNAs). Columns and rows represent samples and miRNAs, respectively.

RNA extracted from omentum-adherent tumors of human IGROV-1 ovarian carcinoma-bearing mice treated i.p. with CpG-ODN or saline (control group) as described [3] was analyzed for miRNA expression using Illumina human miRNA_v2 array. Out of 1145 miRNAs represented on the Illumina chips, 567 mature miRNAs annotated on miRBase12.0, along with 150 putative miRNAs, were consistently detected. Class comparison identified 23 miRNAs showing a FDR<0.1 and a fold-change >1.8 between CpG-ODN- and saline-treated mice (Fig. 1). Among them, 20 miRNAs (16 up-regulated in saline- and 4 in CpG-ODN-treated mice) were annotated on miRBase12.0, whereas 3 miRNAs were putative miRNA sequences derived from deep- sequencing approaches as referred by Berezikov et al. [24] and Solexa (Illumina), which were excluded from further analysis due to the lack of information.

**Figure 2:**
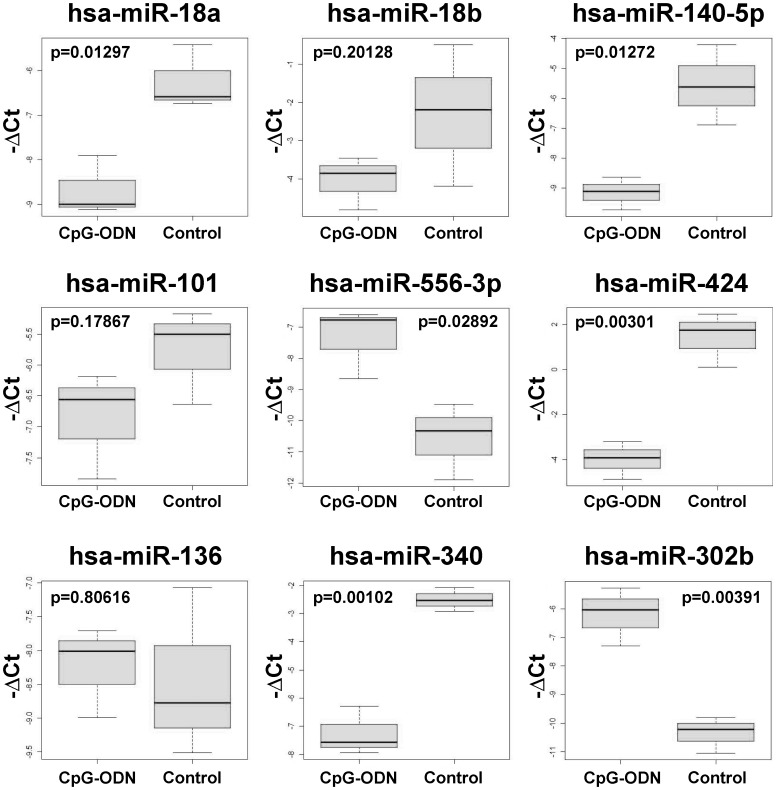
Independent biological validation of CpG-ODN miRNA profile. miRNA expression was assessed by RT-qPCR on IGROV-1 xenografts collected from a replica of a previous experiment [3]. RT-qPCR data are plotted as −ΔCt. P-values were calculated using two-tailed Student’s t-test.

To validate the microarray data, 9 differentially expressed miRNAs were analyzed by RT-qPCR using both the RNA profiled in microarray analysis and the RNA extracted from tumor samples obtained from a replica of the IGROV-1 tumor-bearing mice treated as described [3]. Of the 9 miRNAs, hsa-miR-18a and hsa-miR-18b were selected based on their reported role in the pathogenesis of ovarian cancer [25]; [26], and hsa-miR-101 and hsa-miR-302b for their described involvement in DNA repair processes and sensitivity to chemotherapy [20]; the remaining 5 miRNAs were randomly selected. RT-qPCR using the RNA profiled in microarray analysis validated all 9 miRNAs (Fig. S1), whereas RT-qPCR using the RNA of the replica confirmed 6 of 9 miRNAs (p<0.05), with a trend observed for hsa-miR-18b and hsa-miR-101 but not for hsa-miR-136 (Fig. 2).

### Increased Expression of hsa-miR-302b in IGROV-1 Cells Significantly Improved Cisplatin Activity

**Figure 3:**
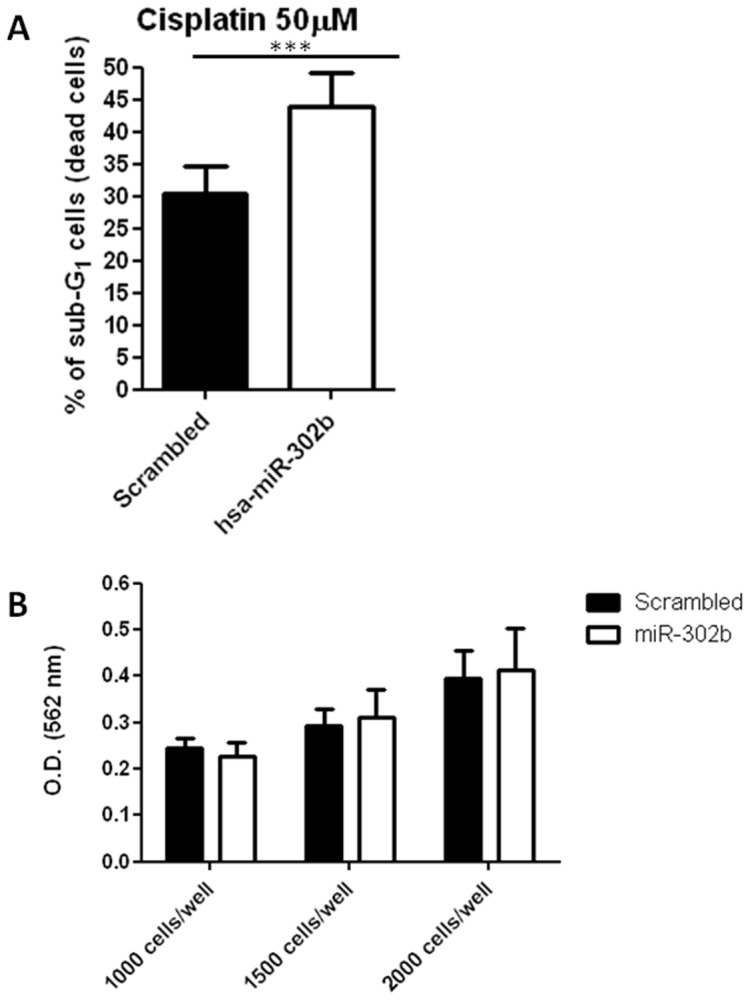
Forced expression of hsa-miR-302b increased cisplatin sensitivity in IGROV-1 cells without affecting cell proliferation. (A) Percent cell death of hsa-miR-302b- and scrambled-transfected cells after cisplatin treatment. IGROV-1 cells were transfected with 50 nmol/l hsa-miR-302b precursor molecule or scrambled control, and 72 h later, exposed to cisplatin (50 µM) for 1 h. Cell viability was assessed 24 h after cisplatin treatment by propidium iodide staining and flow cytometry. Data represent mean ± SEM of 6 independent experiments. ***p<0.0001 by paired t-test. (B) Evaluation of cell proliferation by SRB assay. Transfected cells were seeded in a 96-well plate at a density of 10^3^, 1.5×10^3^, and 2×10^3^ cells/well. Cell growth was assessed by optical density (OD) determination 72 h after transfection. Data represent mean ± SEM of 3 independent experiments.

We previously showed that TLR9-expressing cells in the tumor microenvironment can sensitize cancer cells to DNA-damaging cisplatin treatment by down-modulating genes involved in DNA repair [3]. To determine whether miRNAs modulated by CpG-ODN treatment are able to modify the sensitivity to DNA-damaging agents, the 3 most significantly differentially expressed miRNAs in tumor samples obtained from the replica of the *in vivo* experiment (hsa-miR-424, hsa-miR-340 and hsa-miR-302b) were examined applying a gain- or loss-of-function phenotype in order to mimic the up- or down-modulation observed in miRNA profiling (see Fig. 1). To reduce expression of hsa-miR-424 and hsa-miR-340 (down-modulated in our miRNA expression profile), IGROV-1 cells were transiently transfected for 72 h with the respective LNA inhibitors or with a LNA negative control, whereas cells were transfected with hsa-miR-302b precursor molecule (or a scrambledoligonucleotide) to increase expression of hsa-miR-302b. Cells were then treated with 50 µM of cisplatin for 1 h and after 24 h the percentage of sub-G_1_ cells (as an indicator of cell death) was determined by flow cytometry. Reduced expression of hsa-miR-424 or hsa-miR-340 did not significantly improve cisplatin cytotoxicity (data not shown), whereas increased expression of hsa-miR-302b significantly enhanced cisplatin cytotoxicity, with an increase of cell death ranging from 26.5 to 43.9% in 6 independent experiments as compared to negative scrambled-transfected cells (p<0.0001; Fig. 3A). No significant differences in cell growth were observed between IGROV-1 cells transiently transfected with hsa-miR-302b precursor molecule and control cells (Fig. 3B), ruling out the possibility that hsa-miR-302b sensitized cancer cells to cisplatin by stimulating cell proliferation.

### HDAC4 is Directly Targeted by hsa-miR-302b in IGROV-1 Cells

Because the effects of miRNAs might lead to expression changes in their predicted target genes, we searched for expression patterns deregulated following CpG-ODN treatment by integrating the miRNA and mRNA expression profiles. Class comparison of whole gene expression, previously identified by Illumina HumanHT12_v3 beadchips using the same RNA samples assessed for miRNA profile [3], revealed 215 genes differentially expressed (141 up-regulated in saline- and 74 in CpG-ODN treated mice; FDR<0.1 and fold change >1.8).

**Figure 4:**
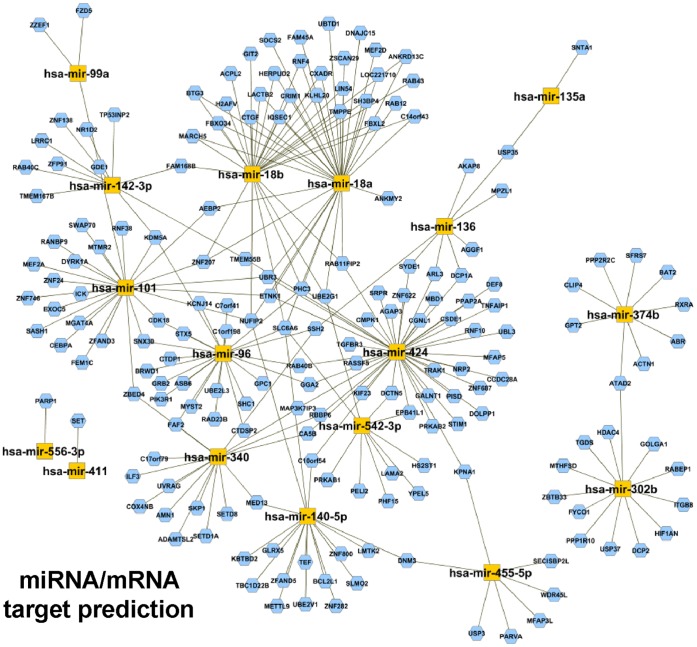
Computational integration of miRNA and gene expression profiles of tumor samples from CpG-ODN- and saline-treated mice. Network between 15 of 20 differentially expressed miRNAs and their anti-correlated target genes. The top 250 interactions were used to generate the network using the MAGIA tool.

To identify functional miRNA-mRNA relationships, miRNA and mRNA data were integrated using the freely available tool MAGIA [19]. Given the frequent miRNA-miRNA interactions, the 20 miRBase-annotated miRNAs were altogether compared to the gene expression profile dataset using the union of Pita, miRanda and TargetScan prediction target algorithms available on MAGIA. The Pearson’s correlation between each miRNA and its predicted target was then computed. The first 250 most significantly negatively correlated miRNA-mRNA interactions were visualized as a network using Cytoscope. Evidence of the concerted interplay of miRNAs regulated by CpG-ODN and their potential target mRNAs was observed (Fig. 4) for 2 miRNAs upregulated (hsa-miR-302b and hsa-miR-374b) and for 13 miRNAs downregulated in CpG-ODN-treated mice (hsa-miR-135a, hsa-miR-136, hsa-miR-340, hsa-miR-445-5p, hsa-miR-424, hsa-miR-96, hsa-miR-142-3p, hsa-miR-140-5p, hsa-miR-542-3p, hsa-miR-18a, hsa-miR-18b, hsa-miR-101, and hsa-miR-99a). The latter 13 form a highly interconnected cluster where different miRNAs exert their biological functions targeting the same genes.

**Figure 5:**
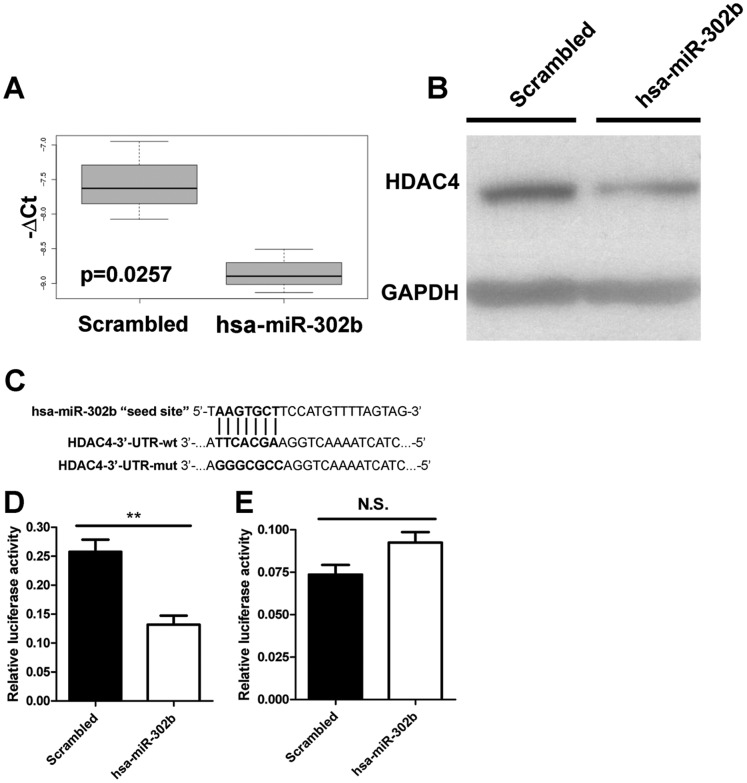
Targeting of HDAC4 in IGROV-1 cells by hsa-miR-302b. IGROV-1 cells were transfected with 50 nmol/l hsa-miR-302b or a scrambled oligonucleotide and RNA and proteins were collected after 72 h. HDAC4 mRNA levels were evaluated by RT-qPCR (A) and protein expression was evaluated by Western blot (B). GAPDH was used to normalize protein loading per lane. Data are representative of 6 independent experiments with superimposable results. (C) Schematic representation of the interaction between hsa-miR-302b and the binding site on the wild-type HDAC4-3′UTR and the mutated control. (D) Relative luciferase activity in IGROV-1 cells for HDAC4-3′UTR-wt co-transfected with reporter vector and with hsa-miR-302b precursor molecule or negative scrambled control for 48 h. (E) Relative luciferase activity in IGROV-1 cells for HDAC4-3′UTR-mut co-transfected with reporter vector and with hsa-miR-302b precursor molecule or negative scrambled control for 48 h.

Focusing on the 19 genes potentially targeted by hsa-miR-302b as identified using MAGIA (q value <0.1, Table S1), we evaluated HDAC4, one of the top anti-correlated mRNAs, as a potential molecular target of hsa-miR-302b associated with response to chemotherapy. HDAC4, a member of the histone deacetylase family, encodes a protein that reportedly mediates cisplatin sensitivity in ovarian cancer [27]. Forced hsa-miR-302b expression in IGROV-1 cells decreased HDAC4 mRNA and protein levels (Fig. 5A and B), supporting the interaction analysis data. To determine whether the down-modulation of HDAC4 after hsa-miR-302b overexpression was due to a direct interaction between the miRNA and the mRNA of HDAC4, a luciferase reporter assay was performed. Briefly, the target site of hsa-miR-302b was identified within the HDAC4 3′UTR according to the Target Scan database (Fig. 5C), and the region including this site was cloned downstream of the luciferase gene into the reporter plasmid pGL3 control. Analysis of IGROV-1 cells co-transfected with hsa-miR-302b precursor or a scrambled oligonucleotide and the reporter vector, containing HDAC4 3′UTR, revealed a significant decrease in luciferase activity in hsa-miR-302b transfected cells as compared to scrambled transfected cells (∼50% reduction, p = 0.0088, Fig. 5D), whereas mutated HDAC4-3′UTR escaped this inhibition (Fig. 5E). These data indicate the direct effect of hsa-miR-302b on HDAC4 gene expression.

### CpG-ODN-modulated miRNAs and Ovarian Cancer Patients’ Clinical Course

**Figure 6:**
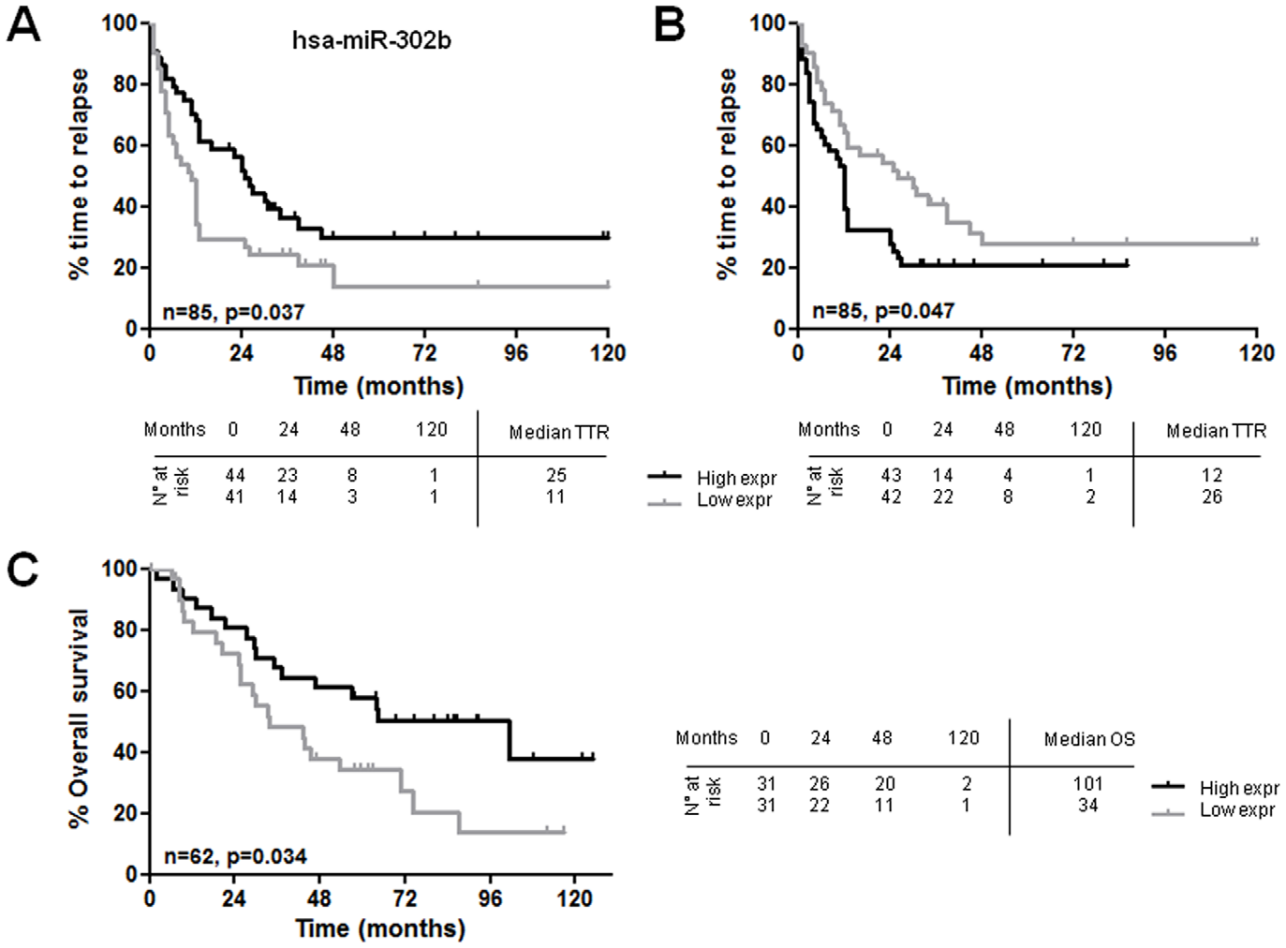
In silico evaluation of ovarian cancer patients’ clinical course according to hsa-miR-302b and hsa-miR-340 expression levels. Kaplan-Meier survival curves of patients stratified according to hsa-miR-302b expression (A) and has-miR-340 expression (B) on GSE25204 and referred to TTR. (C) Kaplan-Meier survival curves for hsa-miR-302b expression on GSE27290 and referred to OS. Patients were dichotomized using median expression as threshold.

The impact of expression levels of all 20 differentially expressed miRNAs, including those for which validation was not carried out, on the clinical course of ovarian cancer patients undergoing chemotherapy was evaluated *in silico*. The time to relapse (TTR) and overall survival (OS) with respect to each miRNA on two public datasets (GSE25204 and GSE27290) [21]; [22] were analyzed. Patients were stratified according to miRNA expression below (low expression) or above (high expression) the median expression value. In Bagnoli’s dataset [22], Kaplan-Meier analysis showed that patients with low expression of hsa-miR-302b or with high expression of hsa-miR-340 had a shorter TTR (log-rank, P = 0.037; HR = 1.75, 95% CI: 1.03–2.95 and P = 0.047; HR = 1.7, 95% CI: 1.01–2.86, respectively) (Fig. 6A and 6B). Median TTR was 11 and 25 months for low and high expression of hsa-miR-302b (Fig. 6A), and 26 and 12 months for low and high expression of hsa-miR-340, respectively (Fig. 6B). In Shih’s dataset [21], only the expression of hsa-miR-302b was significantly associated to OS (log-rank, P = 0.034; HR = 2.02, 95%CI: 1.05–3.88), with a median OS of 33.7 and 101.2 months for low and high expression, respectively (Fig. 6C). In both datasets, the impact of the other 18 miRNAs expression was not significantly associated to TTR or OS (data not shown).

## Discussion

miRNAs are a ubiquitous feature of all cells, and functional studies prompted by the growing number of miRNA targets identified have demonstrated the involvement of miRNAs in the regulation of almost every cellular process investigated, including development, proliferation, differentiation, apoptosis, and tumorigenesis [28]. Moreover, emerging evidence suggests that miRNAs play important roles in the regulation of immunological functions, including innate immune responses of macrophages and the development, differentiation, and function of T and B cells [29]; [30].

Changes in miRNA expression induced by TLR ligand stimulation have been broadly investigated for their impact on development and function of innate immune cells, the primary expressors of TLRs [31]. Here, we show that *in vivo* treatment with TLR9 agonist CpG-ODNs also induces modulation of several miRNAs in tumor cells. This modulation is unlikely to reflect a direct action of CpG-ODN on IGROV-1 cells, which do not express TLR9 and do not respond to murine CpG-ODN, and instead is likely mediated by TLR9-positive cells in the tumor microenvironment directly and/or through soluble factors. Moreover, several studies indicating that miRNAs can also be transferred between cells, e.g., through exosomes [32], as a mechanism to interact and exchange information raises the intriguing possibility that the immune system responds to CpG-ODN treatment by boosting miRNA modulation and interaction with tumor cells.

Our analysis of 3 miRNAs (hsa-miR-424, hsa-miR-340 and hsa-miR-302b) for their relevance to chemotherapy response showed that the enforced expression of hsa-miR-302b on IGROV-1 cells significantly enhanced cisplatin cytotoxicity. Consistent with *in vitro* data, hsa-miR-302b expression was significantly associated to TTR or OS in two datasets of ovarian cancer patients treated with platinum-based therapy. These findings indicate that hsa-miR-302b acts as a “chemosensitizer” in human ovarian carcinoma cells and may represent a biomarker able to predict response to cisplatin treatment, leading to a more accurate selection of patients potentially responsive to a specific therapy.

Moreover, the correlation between miRNA expression and response to specific therapies also suggests the potential usefulness of miRNAs as therapeutic adjuvants. Notably, whereas to date this hypothesis mainly derives from *in vitro* gain- or loss-of-function studies, where candidate miRNAs are identified in tumor cell lines with different degrees of sensitivity to specific therapeutic drugs and then targeted in order to overcome drug resistance, by contrast our study starts using an *in vivo* model to select a candidate miRNA, then validated *in vitro* as adjuvant tool and in human samples as predictive biomarker.

The integration of miRNA and mRNA expression profiles upon CpG-ODN treatment revealed a broad concerted interplay of miRNAs with their predicted target mRNAs, suggesting a relevant role for miRNAs in CpG-ODN-induced expression of genes involved in different cellular pathways. Concerning genes involved in DNA repair, miRNA-mRNA interaction analysis identified HDAC4 as a gene potentially targeted by hsa-miR-302b, as then validated by the decreased HDAC4 mRNA and protein levels upon enforced hsa-miR-302b expression in IGROV-1 cells. Inhibition of HDAC has been reported to induce hyperacetylation of core histones and consequent relaxation of chromatin structure; such an open chromatin configuration would be expected to increase accessibility of genomic DNA to drugs targeting DNA [33]; [34]. These data have led to clinical studies using HDAC inhibitors in combination with current DNA damaging agents, such as topoisomerase inhibitors, DNA synthesis inhibitors, DNA intercalators and agents that covalently modify DNA, as treatment of several types of cancer [34]; [35]. However, whereas clinical studies have shown efficacy against human hematologic malignancies, results in solid tumor trials have been unsatisfactory because of some HDAC inhibitor limitations such as cardiac toxicity [36]; [37]. The observation that overexpression of miR-302b increased the sensitivity of ovarian tumor cells to cisplatin, together with the reported tissue specificity of miRNAs [38], raises the possibility of using this miRNA to modulate DNA-damaging drug sensitivity and avoiding HDAC inhibitor toxicity. Moreover, a very recent study reports direct regulation of p21 protein by members of the miR-302 family activated following DNA damage in human embryonic stem cells [20], further suggesting that miR-302 can impact the response to DNA-damaging agents by modulating different target molecules.

Overall, miRNA modulators are no longer merely theoretical, as evidenced by the recent demonstration that inhibition of hsa-miR-122 reduces viral load in hepatitis C patients [39], and instead are strong candidates as therapeutic agents [39].

## Supporting Information

Figure S1 **qRT-PCR validation of CpG-ODN miRNA profile.** Comparison of hsa-miR-18a, hsa-miR-18b, hsa-miR-140-5p, hsa-miR-101, hsa-miR-556-3p, hsa-miR-424, hsa-miR-136, hsa-miR-340, hsa-miR-302b expression obtained by miRNA expression profile and qRT-PCR on tumors collected from human IGROV-1 ovarian tumor-bearing mice treated daily i.p. with CpG-ODN or saline (control group). *P* values of differential expression between control and CpG-ODN-treated IGROV-1 xenografts are reported. qRT-PCR data are plotted as -ΔCt and array data are plotted as log_2_ (expression).
(TIF)

Table S1 **MAGIA output representing 699 interactions between miRNA and mRNA of CpG-ODN-treated tumors with q value<0.1.**
(XLSX)

Materials & Methods S1 **Supporting materials and methods.**
(DOCX)
