# Supplementary figures and images for: Increased Sensitivity to Chemotherapy Induced by CpG-ODN Treatment Is Mediated by microRNA Modulation
Source: PLoS One. 2013 Mar 6;8(3):e58849. doi: 10.1371/journal.pone.0058849 (PMC3590172; doi:10.1371/journal.pone.0058849)

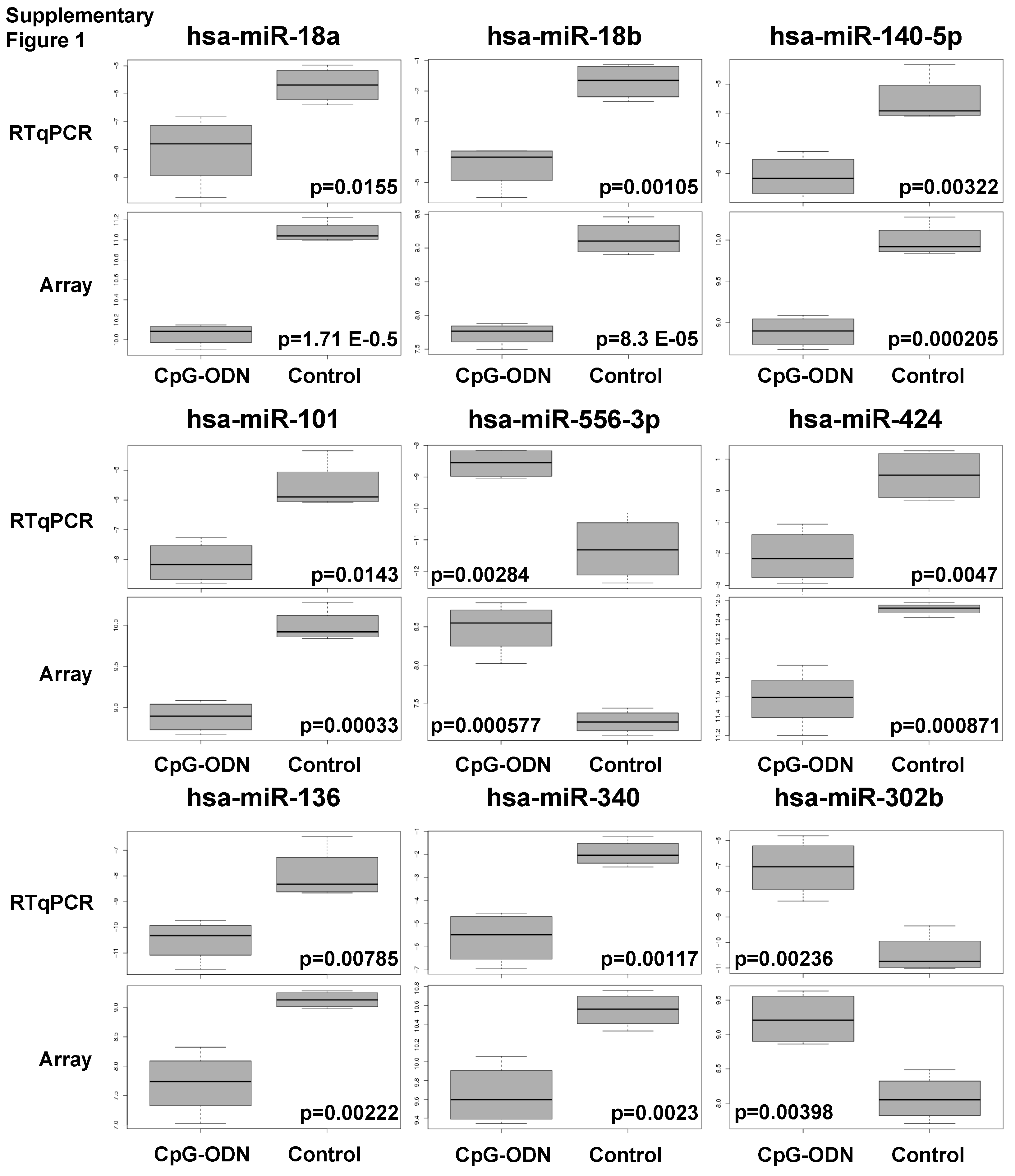

Supplement: Figure S1 — qRT-PCR validation of CpG-ODN miRNA profile. Comparison of hsa-miR-18a, hsa-miR-18b, hsa-miR-140-5p, hsa-miR-101, hsa-miR-556-3p, hsa-miR-424, hsa-miR-136, hsa-miR-340, hsa-miR-302b expression obtained by miRNA expression profile and qRT-PCR on tumors collected from human IGROV-1 ovarian tumor-bearing mice treated daily i.p. with CpG-ODN or saline (control group). P values of differential expression between control and CpG-ODN-treated IGROV-1 xenografts are reported. qRT-PCR data are plotted as -ΔCt and array data are plotted as log2 (expression). (TIF) [file pone.0058849.s001.tif]
