# Supplementary material for: Increased Sensitivity to Chemotherapy Induced by CpG-ODN Treatment Is Mediated by microRNA Modulation
Source: PLoS One. 2013 Mar 6;8(3):e58849. doi: 10.1371/journal.pone.0058849 (PMC3590172; doi:10.1371/journal.pone.0058849)
Supplement: Materials & Methods S1 — Supporting materials and methods. (DOCX) [file pone.0058849.s003.docx]

**Supplementary Material and Methods**

### Drugs and antibodies

Purified phosphorothioated ODN1826 (5’-TCCATGACGTTCCTGACGTT-3’) containing CpG motifs was synthesized by TriLink Biotechnologies (San Diego, CA, USA). Phosphorothioate modification was used to reduce susceptibility of the ODN to DNase digestion, thereby significantly prolonging its *in vivo* half-life. Cisplatin was purchased from Teva Italia (Milan, Italy). Anti-HDAC4 (D15C3), anti-p21 (sc-397) and anti-GAPDH (GAPDH-71.1) antibodies were purchased from Cell Signaling Technology (Danvers, MA, USA), Santa Cruz Biotechnology (Santa Cruz, CA, USA) and Sigma (St. Louis, MO, USA), respectively.

## miRNA expression profiling

Mature miRNAs were detected with the Illumina Human_v2 MicroRNA expression profiling kit, based on the DASL (cDNA-mediated Annealing, Selection, Extension, and Ligation) assay, according to the manufacturer’s instructions (Illumina Inc., San Diego, CA, USA). Briefly, 600 ng/sample total RNA was converted to cDNA followed by annealing of a miRNA-specific oligonucleotide pool consisting of: i) a universal PCR priming site at the 5' end; ii) an address sequence complementary to a capture sequence on the BeadArray; and iii) a miRNA-specific sequence at the 3' end. After PCR amplification and fluorescent labeling, probes were hybridized on Illumina miRNA BeadChips, washed, and fluorescent signals were detected by the Illumina BeadArrayTM Reader. Data were collected using BeadStudio V3.0 software.

**Real-time quantitative PCR (RT-qPCR)**

RT-qPCR microRNA assays specific for hsa-miR-18a, hsa-miR-18b, hsa-miR-140-5p, hsa-miR-101, hsa-miR-556-3p, hsa-miR-424, hsa-miR-136, hsa-miR-340, hsa-miR-302b were purchased from Exiqon (Vedbaek, Denmark). RT-qPCR was performed using the miRCURY LNA Universal RT microRNA PCR system (Exiqon) according to the manufacturer’s instructions. Total RNA (20 ng) was polyadenylated and reverse-transcribed at 42°C (60 min), followed by heat-inactivation at 85°C (5 min) using a poly-T primer containing a 5’ universal tag. The resulting cDNA was diluted 80-fold and 8 μl used in 20-μl PCR amplification reactions at 95°C for 10 min, 40 cycles of 95°C for 10 sec, and 60°C for 60 sec. Results were normalized with snord48 (Assay ID:203903). P-values were calculated using two-tailed Student’s t-test.

### Cell growth assay

### IGROV-1 cells were transfected with 50 nmol/l pre-hsa-miR-302b or scrambled oligonucleotide using SiPort Neo-FX transfection reagent according to the manufacturer’s protocol (Ambion) and seeded in a 96-well plate at a density of 10^3^, 1.5 x 10^3^, and 2 x 10^3^ cells/well. After 72 h of culture, cells were fixed with 10% trichloroacetic acid for 1 h at 4°C, washed 5 times with distilled and de-ionized water, air-dried, and incubated with 100 μl sulforodamine (SRB) 0.4% (w/v) for 30 min. Cells were then washed 4 times with 1% acetic acid, air-dried, and 10 mM Tris solution (pH 10.5) added to dissolve the bound dye. Cell growth was assessed based on optical density (OD) at 550 nm using an ELISA microplate reader (Bio-Rad Lab, Inc., Hercules, CA, USA).

**Immunoblotting**

Transfected cells were lysed in lysis buffer containing 50 mM Tris-HCl (pH 7.5), 150 mM NaCl, 1% Triton X-100 (Sigma), 10% (vol/vol) glycerol, 2 mM Na-orthovanadate, 10 mM leupeptin, 10 mM aprotinin, 1 mM phenylmethylsulfonyl-fluoride, 100 mM Na-fluoride, and 10 mM Na-pyrophosphate for 30 min at 4°C. Insoluble material was removed by 10-min centrifugation at 15,500 × g at 4°C. Protein concentrations were determined using the Coomassie technique. Equal amounts of total lysates (20 μg) were loaded and separated on 10% precast NuPage SDS-Bis-Tris gels (Invitrogen) and transferred to PVDF membranes (Millipore, Billerica, MA, USA). Western blots were performed with the indicated antibodies, and binding was detected with peroxidase-conjugated secondary antibodies and chemiluminescence ECL (GE Healthcare, Little Chalfont, UK) according to the manufacturer's instructions. Quantitation of p21 protein levels reportedly regulated post-transcriptionally by miRNAs of the hsa-miR-302 family (23) revealed the expected down-regulation of this protein in IGROV-1 cells transfected with hsa-miR-302b precursor (data not shown), suggesting that hsa-miR-302b exerts biological effects in IGROV-1 cells similar to those observed in other cellular models.

**Plasmid construction**

For luciferase reporter experiments, a 1017-bp region of the HDAC4 3’ untranslated region including the binding site for hsa-miR-302b was amplified from IGROV-1 cells. The PCR product was digested with XbaI and cloned into the reporter plasmid pGL3 control (Promega, Madison, WI, USA) downstream of the luciferase gene.

Primers for plasmid construction were:

HDAC4 Fw: 5’-AATTTCTAGAGGGGGACTTAATTCTAATCTCATT-3’

HDAC4 Rw: 5’-AATTTCTAGATTTTGTGTCAGACCATTACGAA-3’

**Luciferase assays for target and promoter identification**

pGL3 reporter vector (200 ng) containing the hsa-miR-302b binding site, 40 ng of the phRL-SV40 control vector (Promega), and 50 nmol/l miRNA precursors or scrambled sequence miRNA control (Ambion Inc, Austin, TX. USA) were co-transfected into IGROV-1 cells in 48-well plates. Cells were transfected with Lipofectamine 2000 (Invitrogen) according to the manufacturer’s instructions. Firefly luciferase activity was measured with a Dual Luciferase Assay Kit (Promega) 48 h after transfection and normalized with a Renilla luciferase reference plasmid. Reporter assays were carried out in quadruplicate. Data (mean±S.E.M.) were analyzed using unpaired Student‘s t-test.
